# The role of dual antiplatelets in geographic atrophy secondary to non-neovascular aged-related macular degeneration

**DOI:** 10.3389/fopht.2022.984903

**Published:** 2022-09-08

**Authors:** Yodpong Chantarasorn, Warin Smitthimathin, Pongpat Vorasayan

**Affiliations:** ^1^ Department of Ophthalmology, Vajira Hospital, Navamindradhiraj University, Bangkok, Thailand; ^2^ Department of Ophthalmology, Metta Pracharak Hospital, Ministry of Public Health, Nakhon Pathom, Thailand; ^3^ Neurology Unit, Department of Medicine, King Chulalongkorn Memorial Hospital, Bangkok, Thailand

**Keywords:** non-neovascular AMD, geographic atrophy, pharmacologic (drug) therapy, cRORA, antiplatelets, drusen, pachychoroidopathy

## Abstract

**Background:**

To evaluate the effects of dual antiplatelets on progression of geographic atrophy (GA) secondary to age-related macular degeneration (AMD), and to determine additional factors predicting rapid GA growth.

**Material and Methods:**

In this retrospective cohort study, patients with unifocal GA were consecutively enrolled (one eye per patient) from 2018 to 2021. The patients were categorized as 1. those receiving dual antiplatelet therapy containing a daily dose of 75 mg clopidogrel plus 81 mg aspirin (DAPT group), and 2. those not receiving DAPT (control group). Areas of GA, based on red-filtered fundus autofluorescence, were measured at baseline, and at 3, 6, and 12 months. The primary outcome was absolute 12-month changes in the square root (SQRT) area.

**Results:**

One eye in each group developed neovascular AMD and was excluded from the analysis. The DAPT (24 eyes) and control (22 eyes) groups had comparable age and baseline SQRT area (1.2 ± 0.27 and 1.8 ± 0.41 mm, respectively; *p* adjusted for age = 0.23). At 12 months, after controlling for age and the presence of soft drusen or reticular pseudodrusen, patients receiving DAPT had fewer changes in the SQRT area than that of the control group (0.097 vs. 0.17 mm; *p* = 0.02). The presence of drusen significantly predicted increased GA growth and choroidal thickness reduction.

**Conclusions:**

Routine uses of dual antiplatelets were associated with decelerating GA growth. Drusen-associated GA may represent a generalized form of choroidal vascular alterations.

## Introduction

Geographic atrophy (GA) secondary to age-related macular degeneration (AMD) is a leading cause of irreversible visual impairment worldwide (1, 2). Although the clinical appearance of GA can vary from patient to patient, they do share some characteristics, including a progressive loss of the retinal pigment epithelium (RPE) accompanied by atrophy of adjacent photoreceptors and underlying choriocapillaris (3). No approved treatment is currently available for this condition, but there are several drugs targeting the complement factor cascade in ongoing clinical trials (4). While the pathophysiology of GA is not fully elucidated, its occurrence is considered one of the hallmarks of advanced stage AMD, which is an etiologically complex disease comprising numerous factors, such as inflammation, photooxidative stress, aberrant metabolism, and genetic factors (5–8).

In recent years, increasing evidence suggests that GA could arise from choriocapillaris ischemia (9, 10), whereas vascular endothelial growth factor (VEGF)-driven choroidal neovascularization (CNV) is the compensatory mechanism stimulated by such vascular insufficiency (11–14). This possible AMD pathogenesis has been supported by many studies. First, several studies have identified choroidal thinning and impaired choriocapillaris flow both inside and surrounding the atrophic RPE by optical coherence tomography (OCT) angiography (**Supplementary Figure S1**). The degree of such flow deficits reportedly correlates with the GA expansion rates (10, 15, 16). Second, the presence of concurrent CNV, and the slight degree of persistent subretinal fluid have been shown to be protective against GA progression (17–20). Third, focal choroidal excavation, a pathologic entity that presumably results from choriocapillaris dropout, frequently has co-localization with subsequent pathology including CNV (17, 21). Finally, apart from the AMD spectrum, reticular pseudodrusen (RPD) has exclusively been identified in choroidal infarction secondary to malignant hypertension (**Supplementary Figure S2**) (22); This subretinal pseudodrusen is linked to global choroidal depletion and the development of GA in eyes with AMD (23–25).

Theoretically, several complement factor pathways closely correlate with GA formation (26–28), and play an essential role along with platelets in atherosclerotic plaque, leading to thrombus formation (29, 30). Given that AMD is also associated with pro-atherosclerotic risk factors, regular uses of high-dose antiplatelets in patients with GA may prevent further choriocapillaris occlusion, outer retinal hypoxia, and subsequent RPE degeneration.

In this study, we aimed to determine the effects of dual antiplatelets on the GA progression, and to identify predictive biomarkers responsible for it.

## Material and methods

This observational, retrospective cohort study was conducted at two tertiary centers. The Institutional Review Board approved the study (COA No.095/2564 and the extended protocol No.108/64E), and the principles for this research were based on the Declaration of Helsinki.

Medical records of AMD patients with unifocal GA were consecutively reviewed from August 2018 to June 2021. We excluded patients with the following conditions: multifocal GA, active neovascular AMD, a history of anti-VEGF treatment or macular laser, submacular fibrosis, myopic degeneration, and a concomitant use of anticoagulants. All patients were required to have at least one OCT angiography (PLEX Elite 9000, Zeiss, Germany) scan to exclude the possibility of occult CNV.

In this study, definition of geographic atrophy included 1) color fundus photography (CFP): circumscribed areas of depigmentation of the RPE with a sharply demarcated border between atrophic and non-atrophic retina (31), 2) fundus autofluorescence (FAF): well-defined homogenously hypoautofluorescence areas corresponded to the CFP images. Near-infrared reflectance images could be a supplementary tool to assess lesion boundaries close to the fovea (32), 3) Corresponding OCT images demonstrated a zone of attenuation of the RPE ≥250 µm in diameter in any lateral dimension (33).

Reviewed patients were categorized into two groups: the dual antiplatelet therapy (DAPT) group, consisting of those receiving a daily dose of 75 mg clopidogrel plus 81 mg aspirin, and the control group, which included those whose medication regimen did not involve dual-antiplatelets, or only included 81 mg/day of aspirin. In the DAPT group, we included only patients who had received dual antiplatelets for more than 12 months. The baseline visit was considered the time at which the patients started DAPT or switched from single antiplatelet to DAPT. If the DAPT baseline was undetermined, we retrospectively collected consecutive data from the last 12 months before drug termination (the time patients stopped DAPT), or their most recent visits would be recorded as the study exit (12-month follow-up).

**Figure 1:**
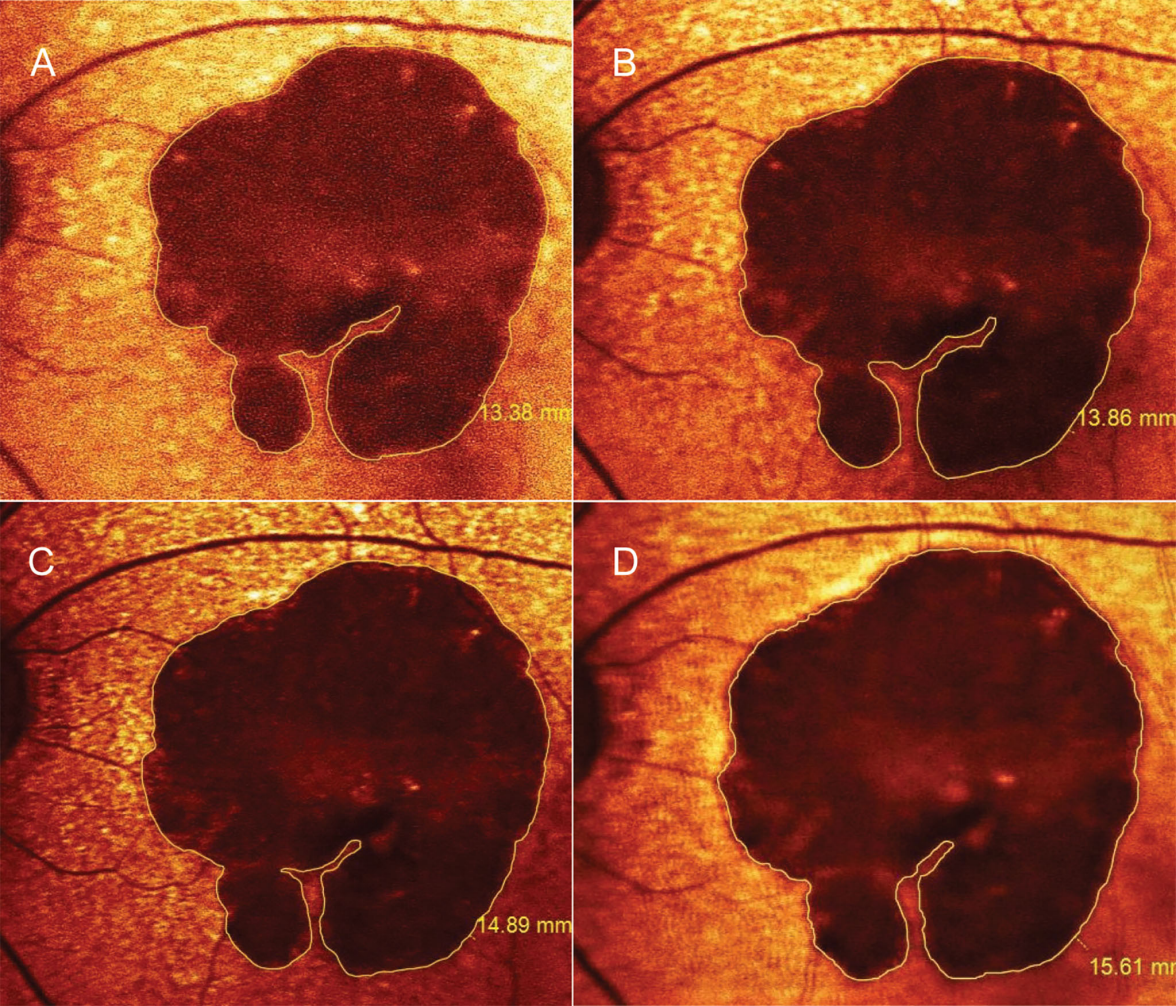
Progression of geographic atrophy (GA) measured by fundus autofluorescence. A 79-year-old woman was diagnosed with horseshoe-shaped GA surrounded by subretinal drusenoid deposits in the left eye. While the GA was slightly enlarged from baseline **(A)** to 12 months **(B)**, her vision remained unchanged at 20/200 over the first year after starting aspirin combined with clopidogrel. The lesion expanded to 14.9 mm^2^ at 3 months **(C)** and to 15.6 mm^2^ at 6 months after drug discontinuation **(D)**; Her visual acuity deteriorated to counting finger along with the loss of inferior parafoveal remnants. Her clinical and imaging course was consistent with treatment effects analyzed in the study patents while on DAPT **(A, B)**. Nonetheless, we did not investigate the late effects appearing after drug discontinuation **(C, D).** Figure 1 without the measurement markers is shown in **Supplementary Figure 4**.

One eye in each patient was selected for the study to minimize the confounding effects acquired from the co-dependent variables, including demographics and systemic vascular diseases. In patients with bilateral GA, the eye with more vision-threatening lesions was selected as the study eye. In particular, eyes with fovea-sparing paracentral GA (**Figure 1**) were chosen instead of those with preexisting large central GA. The criteria were designed to make the study results applicable to clinical practice.

**Figure 2:**
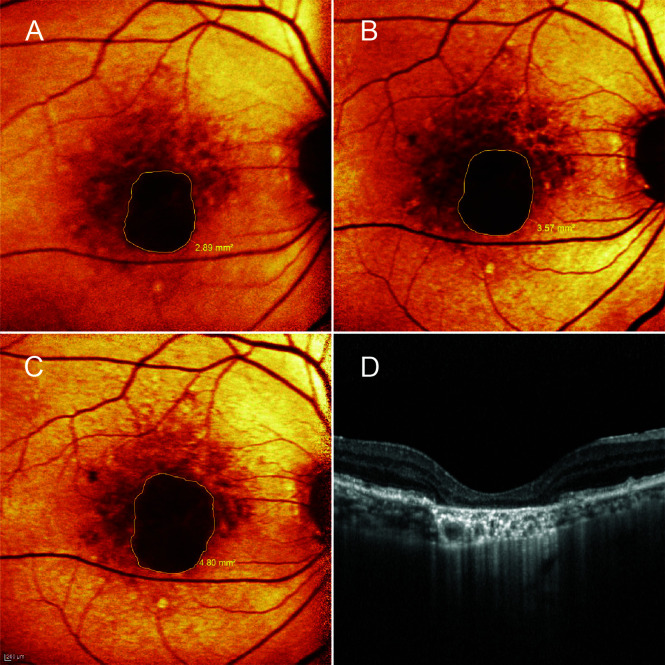
A patient in the control group. A 76-year-old man presented with central geographic atrophy (GA) bordered by soft drusen in the right eye. Fundus autofluorescence showed progressive enlargement of the lesion from baseline **(A)** to 6- **(B)** and 12-month **(C)** visits. At 12 months, the square root GA area had increased from baseline visit by 0.36 mm; A corresponding optical coherence tomography image revealed complete retinal pigment epithelial and outer retinal atrophy (cRORA) coincided with homogeneous choroidal hypertransmission **(D)**. Subfoveal choroidal thickness reduced from 187 µm at baseline to 176 µm at 12-month follow-up.

Areas of GA were manually measured in the red-filtered FAF using the RegionFinder Software (Spectralis OCT2, Heidelberg Engineering). The decision to implement color-coded FAF instead of black-on-white or white-on-black mode was based on general agreement among the authors after reviewing a sample set of FAF images. As a result, red-filtered FAF appeared to be the most effective approach to distinguish atrophic areas from normal-appearing retina (**Figure 1**, **Supplementary Figure S3**). To increase the measurement precision, the thinnest line overlay was selected (**Figure 1**, **Supplementary Figure S4**), and the FAF images were scaled up before measuring lesions. Any ill-defined borders of GA in the FAF were confirmed by a transitional point from intact to disrupted RPE seen on corresponding OCT scans. The OCT findings were graded according to The Consensus Definition for Atrophy Associated with Age-Related Macular Degeneration on OCT (2018) (33). To clarify, the authors classified the GA lesions as incomplete RPE and outer retinal atrophy (iRORA) if there was a persistent hyperreflective line within the bed of atrophy despite a marked attenuation of the RPE band longer than 250 µm. Complete RPE and outer retinal atrophy (cRORA) referred to atrophic lesions with homogeneous choroidal hypertransmission that was present in continuous manner on the OCT (**Figure 2**). Each photo was measured by two masked ophthalmic technicians, and values were averaged across raters.

The square root (SQRT) of the GA area was applied in the analyses to reduce the impact of confounding on the progression rate (34, 35). Absolute 12-month changes in the SQRT GA area were selected as the primary outcome because the values directly correlated with clinical devolution of the central visual field. The parameter has been similarly applied by several clinical trials investigating the efficacy of agents for the treatment of GA (4, 36). In addition, GA growth rate was considered one of the secondary outcome measures. To handle correlated data from repeated measurements of each patient, we performed a linear mixed model analysis of the SQRT GA area, controlled for age and baseline SQRT GA area, to determine differences in the slopes of its expansion rates from months 3 to 12 between the two groups.

A recent study demonstrated that GA coinciding with pachychoroidopathy (pachychoroid GA) was infrequently associated with drusen and grew slower than conventional or drusen-bordering GA (37). Therefore, to investigate predictors of rapid GA expansion, the presence of drusen, either reticular pseudodrusen (RPD) or soft drusen (diameter ≥63 µm), adjacent to macular atrophy was chosen for the covariate adjustment. These two subtypes of drusen have been associated with AMD progression, particularly when combined with pigmentary changes (38). Furthermore, since extrafoveal GA contributes to greater changes in its lesion size compared with central GA, the existence of center-involving GA was included in the baseline comparisons between the two groups (39, 40).

Other secondary outcomes were changes in best-corrected visual acuity (BCVA) and subfoveal choroidal thickness (SFCT) measured by enhanced depth imaging OCT over 12 months. Of note, BCVA was not chosen for the primary endpoint because most GA lesions tended to be foveal sparing (41). The overall retention rate of medications and other standard ocular parameters were obtained at baseline, and at 3, 6 and 12 months.

### Statistical analysis

Details of sample size calculation were described in Method S1. After validation of the implemented model, a multiple regression analysis controlling for age and the presence of drusen-surrounding GA was performed to detect differences in changes in 12-month SQRT GA areas (the primary outcome) between the two groups. Regarding the analysis of SFCT changes, covariate adjustment was implemented by adding potential confounders (age and mean arterial pressure) and significant predictors (the presence of drusen) into the model.

To assess the expansion rates (slopes) of the SQRT GA area, we assumed that the trend over time in both groups was linear. Collected values from each patient were calculated at each follow-up. A crude comparison at each time point was performed using a two-sample t test with unequal variances. A mixed model was applied to correct confounders from repeated measurement in each subject over time. In this model, we allowed the mean values of the intercept and slope to depend on the treatment exposure (DAPT). Additionally, the analysis was controlled for age and baseline SQRT GA area. The two-sided *P* value <0.05 was considered statistically significant. Stata version15.0 (StataCorp, College Station, TX) was used for all computations.

## Results

Among the 96 cases who met the inclusion and exclusion criteria, only 50 had complete imaging data with adequate resolution for assessment. Two patients had poor adherence to antiplatelet medications due to peptic ulcers and were excluded from the analysis. One patient in each group developed subretinal hemorrhage during the study period; both were disqualified from the study because they received anti-VEGF treatment. DAPT was used as thromboembolic prophylaxis after cardiac-bypass (18 of 24 cases, 75%) and ischemic stroke combined with ischemic heart disease (3 cases, 12.5%) or ipsilateral moderate carotid stenosis (3 cases, 12.5%). Three patients (12.5%) in the DAPT group were referred from peripheral hospitals where the records were not available to estimate the time point of starting the medications. The time these patients stopped DAPT was documented as 12-month follow-up visits, and the data drew from the last 12 months before drug termination was analyzed. The overall retention rate of medications over the 12-month cohort was 92% in the DAPT group. Twenty patients in the DAPT group (83%) continued DAPT longer than instructed by their physicians; the mean duration of DAPT was 15.2 ± 4.8 months (range, 13 to 28 months). Four patients (18.1%) in the control group (22 cases) took 81 mg/day of aspirin daily; all four had a treatment duration of less than 4 months during the 12-month study period. Clinical backgrounds and the 12-month study results are detailed in **Tables 1**, **2**, respectively.

**Table 1:** Demographic, concomitant vascular diseases and baseline ocular characteristics of study patients with geographic atrophy.

	DAPT (24 eyes)	Control (22 eyes)	*P*
Demographic			
Age, mean ± SD, y	77 ± 8.2	79 ± 9.9	0.52
Male, No. (%)	13 (54.2)	12 (54.5)	0.87
Baseline Ocular Characteristics, No. (%)			
Increased FAF along the GA borders	15 (62.5)	12 (54.5)	0.67
Presence of soft drusen or RPD adjacent to GA Soft drusen-bordering GA^a^	13 (54.1) 7 (53.8)	11 (50.0) 4 (36.3)	0.81 0.38
Center-involving GA	11 (45.8)	7 (31.8)	0.56
SQRT GA areas, mean ± SD, mm	1.23 ± 0.27	1.88 ± 0.43	0.22^b^
SFCT, mean ± SD, µm	198 ± 19	186 ± 31	0.76^b^
BCVA, mean snellen equivalent ± SD in logMAR BCVA	20/80^-2^ ± 0.40	20/80 ± 0.49	0.88
Concomitant Vascular Risk Factors, No. (%)			
LDL-c to HDL-c ratio, mean ± SD	2.6 ± 1.2	2.1 ± 0.9	0.37
History of smoking > 15 pack-years (%)	9 (37.5)	3 (13.6)	0.16
Use of supplement^c^	3 (12.5)	3 (13.6)	0.85
History of cardiovascular surgery (%)	18 (75)	1 (4.5)	0.02
Cerebrovascular disease (%)	6 (25)	0	NA
Normotensive glaucoma^d^ (%)	3 (12.5)	5 (22.7)	0.38

DAPT, dual antiplatelet therapy; FAF, fundus autofluorescence; GA, geographic atrophy; RPD, reticular pseudodrusen; SQRT, square root; SFCT, subfoveal choroidal thickness; BCVA, best-corrected visual acuity; NA, not applicable; LDL-c, low density lipoprotein-cholesterol; HDL-c, high density lipoprotein-cholesterol; AREDS, age-related eye disease study.

a Analysis of eyes presenting with drusen.

b P value was adjusted by age.

c AREDS2 formula or supplements containing a daily dose of 10mg lutein and 2mg zeaxanthin.

d All was treated with 0.15% topical brimonidine twice daily.

**Table 2:** Changes in macular atrophy areas, choroidal thickness, and visual acuity over 12 months.

	DAPT (24 eyes)	Control (22 eyes)	Adjusted differences (95% CI)	*P* values		Adjusted covariates
				Crude	Adjusted	
SQRT GA areas, mean ± SD, mm						Age
Baseline	1.23 ± 0.27	1.88 ± 0.43	0.54 (-0.37, 1.4)	0.20	0.22	
Months 3	1.27 ± 0.27	1.94 ± 0.42		0.19	0.19	
Months 6	1.30 ± 0.27	1.98 ± 0.41		0.18	0.17	
Months 12	1.33 ± 0.27	2.10 ± 0.41		0.17	0.13	
12-month absolute changes in SQRT GA areas, mean ± SD, mm	0.09 ± 0.08	0.17 ± 0.11	0.07 (0.01, 0.13) 0.06 (0.002, 0.12)	0.06	0.02 0.04	Age, drusen Age, drusen, and baseline SQRT GA area
12-month reduction in SFCT, mean ± SD, µm	1.4 ± 7.7	7.2 ± 9.4	5.7 (-13.3, 24.2)	0.19	0.22	Age, drusen, MAP
12-month mean snellen equivalent ± SD in logMAR BCVA	20/100^+2^ ± 0.41	20/100^+1^ ± 0.55	0.04 (-0.29, 0.38)	0.96	0.79	Center-involving GA
Loss of BCVA, mean ± SD, ETDRS letters	1.8 ± 2.2	3.8 ± 3.7	2.05 (-5.64, 9.75)	0.35	0.29	Center-involving GA

DAPT, dual antiplatelet therapy; SQRT, square root; GA, geographic atrophy; SFCT, subfoveal choroidal thickness; MAP, mean arterial pressure; BCVA, best-corrected visual acuity; ETDRS, early treatment diabetic retinopathy study.

The dual antiplatelets (24 eyes) and control group (22 eyes) had comparable baseline features including age (77 ± 8.2 and 79 ± 9.9 years; *p* = 0.52), lipid profiles (LDL to HDL ratios; 2.6 ± 1.2 and 2.1 ± 0.9; *p =* 0.37), and SQRT GA area adjusted for age (1.23 ± 0.27 and 1.88 ± 0.43 mm; adjusted difference, -0.54 mm; 95%CI, -1.4 to 0.37; *p =* 0.22, respectively) (**Table 1**). Regarding baseline OCT findings, cRORA was identified in 19 (79%) and 15 (68.1%) eyes in the DAPT and control groups, respectively (*p* = 0.76). The remaining study eyes were classified as iRORA. At 12 months, the proportions of eyes with cRORA increased slightly in both groups (20 eyes (83%) and 18 eyes (81.8%) in the DAPT and control groups, respectively) (*p* = 0.44). There were no significant differences regarding the proportions of eyes with center-involving GA between groups (*p* = 0.56).

After controlling for age and the presence of drusen (reticular pseudodrusen or soft drusen), patients receiving DAPT had fewer 12-month changes in SQRT GA area than that in the control group (0.097 vs. 0.17 mm; adjusted difference, 0.07 mm; 95% CI, -0.13 to -0.01 mm; *p =* 0.02). These effects remained significant when the baseline SQRT GA area was included into the model as the third controlled variable (adjusted difference, 0.06 mm; *p* = 0.04) (**Table 2**), which per se did not significantly affect the primary endpoint (estimated coefficients, 0.017 mm; 95%CI, -0.006 to 0.04; *p* = 0.15). No significant interaction between the baseline GA area and use of DAPT was observed in this model (*p* = 0.61).

**Figure 3:**
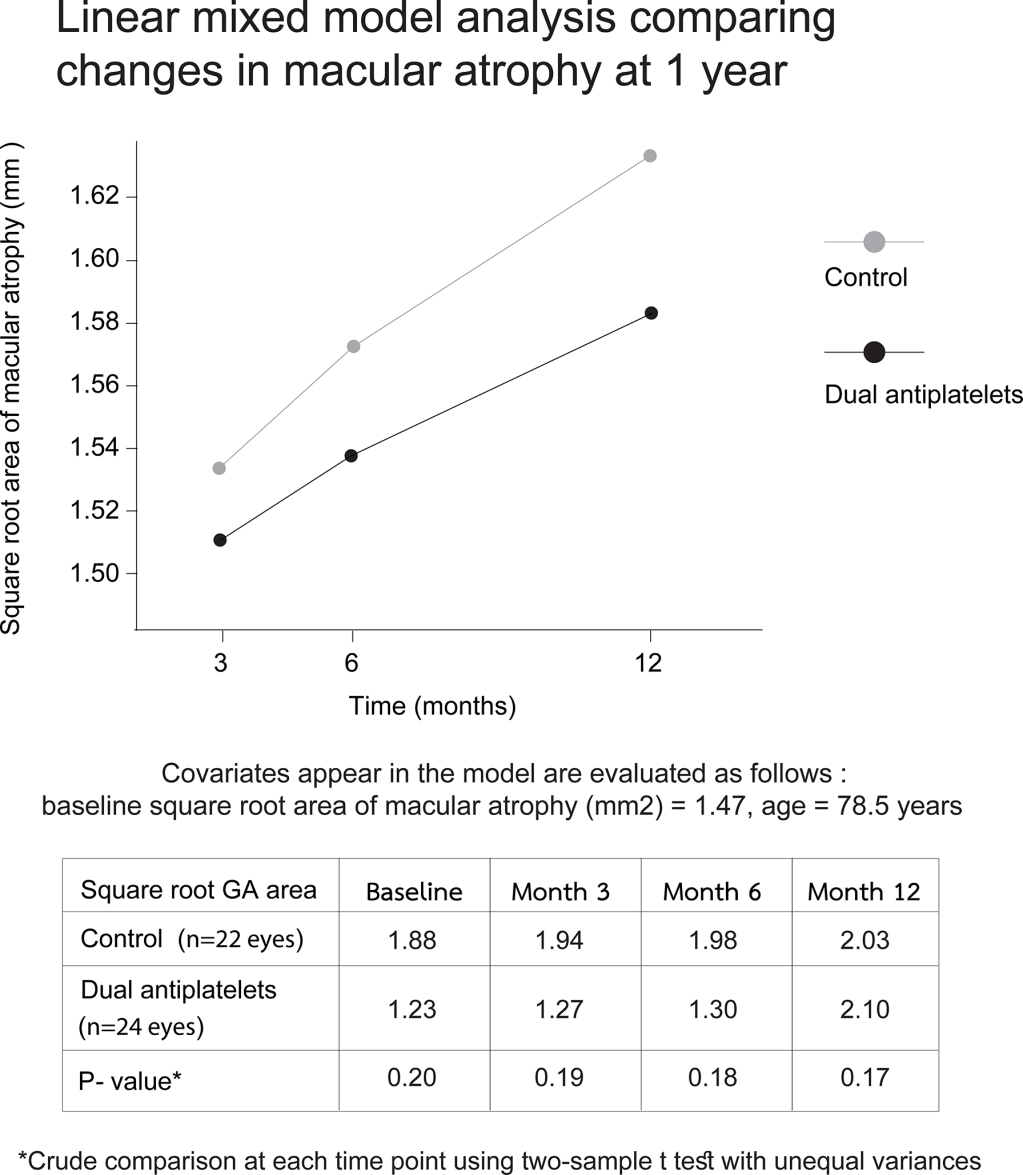
Linear mixed model displaying changes in macular atrophy areas over 12 months. Plots of mixed model analysis, controlling for age and baseline square root area of macular atrophy, demonstrate no significant differences in the overall slope of disease progression between two groups from months 3 to 12 (*p* = 0.16). Each time-point growth-rate comparison shows a gradual divergence with decreasing P-values through time (*p* = 0.19 and 0.17 at month 3 and 12, respectively).

For the secondary outcomes, the linear mixed model analysis, adjusted for age and baseline SQRT GA area, showed no significant differences between the two groups in overall slopes of the GA growth rates from months 3 to 12 (*p =* 0.16). Nevertheless, a divergence of trends with lessening *P*-values was observed over time (**Figure 3**).

Regarding additional factors associated with GA progression, the presence of drusen-bordering GA was the only factor associated with more rapid GA growth (mean 12-month changes in the SQRT GA area, 0.07 ± 0.03 mm; 95% CI, 0.09–0.13; *p =* 0.02). In the subgroup analysis, no significant differences in GA expansion rates were detected between soft drusen versus RPD associated GA (*p* = 0.77) (**Table 2**).

Concerning differences in visual results, the 12-month mean of early treatment diabetic retinopathy study letters lost was comparable between the two groups (1.8 ± 2.2 in the DAPT group, and 3.8 ± 3.7 letters in the control group; *p* adjusted for the presence of center-involving GA = 0.29).

The 12-month mean SFCT reduction in patients receiving DAPT (1.4 ± 7.7 µm) was not significantly different from that in the control group (7.2 ± 9.4 µm) after controlling for age, mean arterial pressure, and the presence of drusen (*p* = 0.22). Conversely, the presence of drusen represented the most important factor predicting mean SFCT loss at all timepoints (difference additionally adjusted for age and baseline SFCT, 24 ± 11 µm per year; *p =* 0.04).

During the study period, none of the patients experienced minor or major bleeding events that led to hemodynamic instability, according to the criteria of the 2020 American College of Cardiology consensus (37).

## Discussion

This study demonstrated that regular uses of a daily dose of 81mg aspirin plus 75mg clopidogrel were associated with less GA expansion over a 12-month observation compared to the control group. To discuss the impact of baseline characteristics, though not statistically significant, mean baseline lesion size in the DAPT group was smaller than that in the control group. This may dispute the main results since preexisting GA size could impact disease progression (41). To reduce such confounding effects, we utilized the square root of the GA area before the analyses; The differences in 12-month GA enlargement between the groups remained significant even when the baseline GA size was included into the preexisting covariate adjustment. In the same model, the effect of DAPT on the primary outcome was not affected by baseline GA size since no significant interaction was observed between these factors (*p* = 0.61). In addition to the primary outcome, the fact that differences in the GA expansion gradient between the groups progressively increased from months 3 to 12 should altogether support the possible effects of the medications on slowing the disease course (**Figure 3**). It is to emphasize that the difference in baseline lesion areas that was not statistically significant could have become significant with a larger cohort. Therefore, the slower disease progression in the DAPT group may not be solely due to the medication effect, but rather could partially result from the consequences of small baseline GA size in those eyes.

Although the exact mechanisms behind our findings cannot be explained by this small observational study, and further prospective studies using OCT angiography in larger number of subjects are required to determine how antiplatelet agents affect choriocapillaris perfusion, it is theoretically possible that antiplatelet drugs may prevent intravascular thrombosis and choroidal ischemia, which has been linked to the AMD pathogenicity (42, 43). The concept has been supported by both histological and *in-vivo* studies pointing that choriocapillaris hypoperfusion may partly be responsible for outer retinal hypoxia during an initial phase of GA; Nearly all retina in postmortem subjects clinically diagnosed with AMD demonstrated a primary breakdown of choriocapillaris despite intact overlying retina and RPE tissues (9). Another histologic study performing in donated human eyes with early AMD demonstrated a significant decrease in density of macular choriocapillaris lobules disproportionate to nearly-normal clinical features on standard *in vivo* imaging (43). Lastly, a previous study utilizing OCT angiography revealed a significant correlation between the GA expansion rate and choriocapillaris flow deficits over the region adjacent to the GA border (16). The magnitude of the choriocapillaris flow void was further translated to the macular function in the same region; This corresponded with a recent observation showing a significant invert correlation between choriocapillaris flow deficits and retinal sensitivity measured by microperimetry at each localized area. Expectedly, choriocapillaris flow and retinal sensitivity improved with distance from the GA border (44, 45).

In physiologic conditions, platelet aggregation is considered a repair response to vascular damage, while a series of exaggerated progressions of such a process can result in intravascular thrombosis and subsequent ischemia. Antiplatelet agents reduce the risk of thrombus formation by interfering with the process of platelet aggregation primarily induced by thromboxane A_2_. Near-complete inhibition of platelet cyclooxygenase (COX)-1, one of the earliest precursors to thromboxane A_2_, can be achieved and sustained with 75-150 mg/day of aspirin (46). Although choriocapillaris is unlikely to represent a primary site of vascular thrombosis due to its fenestrated properties, large-vessel stenosis is often observed in AMD choroid, suggesting that the choriocapillaris blood supply may be limited as well. This potentially contributes to RPE hypoxia and photoreceptor degeneration in the area of hypoperfusion (47, 48). Unfortunately, no histopathologic studies have validated whether vascular alterations in AMD choroid are partly a consequence of platelet-driven mechanisms (49). More importantly, direct visualization of pathogenic arteries behind the choroid is currently not available due to limited imaging technology. Taken together, we can only assume that atherosclerotic changes can occur systematically, and ultimately may result in end-organ hypoperfusion and microcirculatory failure with no exception to the areas of high blood flow circulating in tightly packed capillary wall laid out flat vascular lumens as choriocapillaris.

Unlike inner retinal circulation, choroidal vessels are not autoregulated; Oxygen supply to the outer retina can be depleted by any of its supplied arterial branches with the point of maximum stenosis proximal to the choriocapillaris, such as large choroidal vessels, posterior ciliary artery, ophthalmic artery, or internal carotid artery (50, 51). At present, the effects of various severity of carotid stenosis on GA progression has never been explored; three patients (12.5%) in the DAPT group had a history of cerebral infarction with moderate carotid stenosis. Such alterations in neuro-ophthalmic circulations could influence the choroidal perfusion and possibly accelerate the GA expansion in the DAPT group (52).

In retrospect, patients in the DAPT group had more severe systemic vascular disease than those in the control group, and may have already taken single antiplatelet for a substantial period before switching to DAPT. Therefore, we were unable to determine at baseline whether the small lesion size in the DAPT group was attributable to antiplatelets taken before the study initiation.

Previous studies have indicated that compared to unifocal GA, multifocal atrophic spots in AMD eyes were one of the main features predisposing greater GA enlargement (39). Referring to our study, the control group had mean changes in GA areas of 0.17 mm/year, which was less than the 0.28 mm/year reported in previous studies (34, 53). These results may have been influenced by the study criteria, which only included eyes with unifocal GA.

Regarding the secondary outcomes, global depletion of choroidal vasculature complicating GA was represented by 12-month SFCT reduction (15). The implemented model presented a tendency toward smaller SFCT reduction in the DAPT group compared to that in the control group. However, the presence of drusen-surrounding GA represented a stronger predictor of SFCT loss than antiplatelet use. This may reflect a distinct form of drusen-associated GA where choroidal hypoperfusion appears more generalized than that of drusen-free GA. Most patients with drusen-related macular atrophy typically have a history of macular drusen for many years before the occurrence of GA. This conventional form of GA has been associated with risk factors affecting systemic micro-circulation, which may deteriorate vascular supply to the choroid (54). We speculate that the processes of choriocapillaris ischemia should manifest in a chronic, albeit intermittent, manner due to plethora of collateral circulations in the macular choroid (55).

Notably, apart from retinal ischemia, deposits of drusen material between the RPE basal lamina and the inner collagenous layer of Bruch’s membrane with an eventual formation of drusenoid pigment epithelial detachment (PED) are considered a defined route to GA (56). Such deposits grow slowly but collapse quickly, with a legacy of RPE loss sharply demarcated by the borders of collapsed drusenoid PED. Given the almost identical areas shared by these two entities, the confluence of large drusen can be viewed as a catalyst to the GA occurrence since it intensifies the degenerative process of the RPE cells by blocking their blood supply mismatched to the increasing demand for nutrients and oxygen (38, 57, 58). This altogether may support our results demonstrating that the existence of drusen around the GA border independently accelerated expansion of the SQRT GA area by 0.07 mm per year. Nevertheless, concurrent drusen in atrophic AMD is only one of several factors governing progressive RPE loss because the continuing process of overt GA expansion was present in patients with drusen-free GA observed in this study and those previously reported (37).

Concerning the safety aspects, a previous study highlighted the safety of antiplatelets in AMD by reporting no association between five-year regular use of low-dose aspirin and the incident neovascular AMD (59). Their results are in concert with ours, showing no differences in neovascular complications between the two groups during the study period.

A direct association between neovascular AMD and platelet activation has rarely been explored in the literature. A previous case control study showed that the serum level of platelet-activating factor was significantly higher in patients with polypoidal choroidal vasculopathy compared to that in a control group (30). The Age-Related Eye Disease Study 2 Report No.17 disclosed an inverse correlation between the prevalence of eyes with drusenoid PED and aspirin dosages; Among 325 patients with DPED, 52%, 46.5%, and 1.5% took no aspirin, 81 mg/day aspirin, and ≥160 mg/day aspirin, respectively. Though this was not a primary objective in the study, these findings may support the protective role of antiplatelet drugs against AMD progression (38). In accordance with these studies, platelet-mediated microvascular occlusion at the choriocapillaris level could be metaphorically defined as “choroidal stroke,” which may be a crucial part of the multifactorial and multistep process in AMD. Hence, it may be reasonable to advocate antiplatelet therapy to halt GA progression.

Due to the close relations between ophthalmic and cerebrovascular circulation, elements of well-established evidence on antiplatelet use for secondary prevention of ischemic stroke may be partly applied for similar pathology in the small vessels like choroid (55). In neurology practice, DAPT are recommended in stroke patients with intracranial atherosclerosis with >50% stenosis who had a minor stroke or who have a high risk for transient ischemic attack. It is frequently continued for at least 12 weeks before switching to a single antiplatelet regimen for preventing future cerebrovascular events. However, the exact duration of DAPT is controversial since there is no definite time when acute conditions end (60, 61).

To compare the clinical course with AMD, once GA is established, it will continue to deteriorate in parallel with a life-long depletion of the underlying choroidal vasculature. Thus, the long-term usage of antiplatelets may serve as secondary prophylaxis of progressive choroidal ischemia corresponding with GA. Nonetheless, concerning the fact that long-term use of high-dose antiplatelet may not be suitable for every patient, it would be interesting to conduct a clinical trial investigating the role of low-dose antiplatelets on decelerating macular atrophy secondary to AMD. Obtaining this information may help to develop effective combination therapies, along with complement factor inhibitors, designed to lessen the emergence of choriocapillaris dropouts, and GA progression.

In the present study, six out of 24 patients (25%) in the DAPT group developed cerebral infarction after having been diagnosed with atrophic AMD for several years. Possible common mechanisms for the link between AMD and stroke include, but are not limited to, the complement factor H gene and complement cascade activation in atherosclerotic lesions. Despite some discrepancy on this topic, the relationship with cerebrovascular disease appears more robust in advanced AMD (62–65). Concerning this connection, before initiating antiplatelet therapy in AMD patients who have no history of stroke, brain imaging should be performed to exclude those with preexisting lesions predisposing them to intracerebral hemorrhage.

Our study contains several limitations tied to its retrospective nature. These include small sample size, lack of psychophysical tests, such as microperimetry and low luminance visual acuity (66), and treatment heterogeneity in the control group. However, conducting a prospective, observational study to determine outcomes similar to ours is unlikely to be feasible due to a rare intersection between GA and high-dose antiplatelet usage. Moreover, the follow-up duration was rather short because DAPT is generally tapered to single antiplatelet therapy at one year after cardiovascular intervention. Given the fact that most clinical trials involving GA set the primary endpoints at a two-year follow-up because of slow progressive nature of the disease (4, 36, 54), the 12-month observation may not allow us to establish a significant effect of medications on the progression rates. Finally, the study did not allow us to observe whether a dose-response gradient existed for the observed effect; Such association is believed to act against the biases in non-randomized studies (67).

In summary, during this 12-month cohort, the GA growth appeared to be decelerating in patients who were on continuous dual antiplatelet therapy. Drusen-associated GA may represent a subtype of atrophic AMD that possesses diffuse and more progressive choroidal nonperfusion than GA without drusen. Altogether, the results support that hemodynamic changes in the choroidal vasculature coinciding with outer retinal hypoxia may be associated with atrophic AMD.

## Data availability statement

The original contributions presented in the study are included in the article/**Supplementary Material**. Further inquiries can be directed to the corresponding author.

## Ethics statement

The studies involving human participants were reviewed and approved by The Institutional Review Board of the Faculty of Medicine Vajira Hospital. The patients/participants provided their written informed consent to participate in this study.

## Author contributions

YC and PV designed the study. YC, WS, and PV collected and analyzed the data. YC and PV wrote the manuscript. YC, WS, and PV reviewed and revised the manuscript. All authors contributed to the article and approved the submitted version.

## Funding

The publication charge was supported by Navamindradhiraj University.

## Acknowledgments

The authors would like to thank our ophthalmic technicians, Sinpana Uraruen, B.Sc. and Sirapan Duenthongsuk, B.Sc., for their dedicated efforts on the collection and measurement of GA-related data. We also gratefully acknowledge Assist Prof. Dusit Sujirarat for the data computations and statistical analysis.

## Conflict of interest

YC is a consultant for Bayer, Roche and Novartis.

The remaining authors declare that the research was conducted in the absence of any commercial or financial relationships that could be constructed as a potential conflict of interest.

## Publisher’s note

All claims expressed in this article are solely those of the authors and do not necessarily represent those of their affiliated organizations, or those of the publisher, the editors and the reviewers. Any product that may be evaluated in this article, or claim that may be made by its manufacturer, is not guaranteed or endorsed by the publisher.
