# Supplementary material for: The role of dual antiplatelets in geographic atrophy secondary to non-neovascular aged-related macular degeneration
Source: Front Ophthalmol (Lausanne). 2022 Sep 8;2:984903. doi: 10.3389/fopht.2022.984903 (PMC11182290; doi:10.3389/fopht.2022.984903)
Supplement: Supplementary file 1 [file DataSheet_1.pdf]

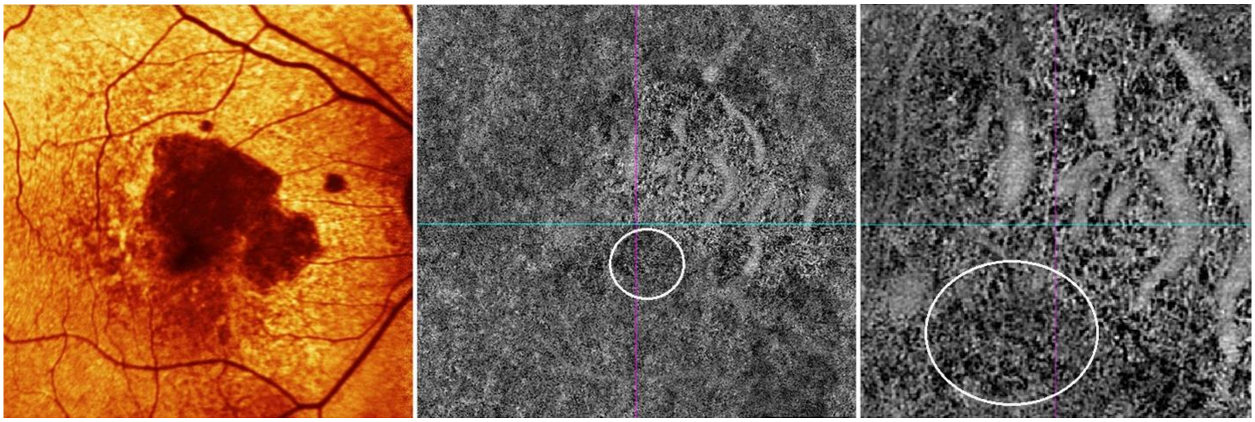

Supplementary Fig. 1. Decreased choriocapillaris density in the area adjacent to geographic atrophy (GA). A reduction in macular autofluorescence was detected inferotemporal to the horseshoe-shaped GA (left panel). Deficits in the choriocapillaris lobules (white circles) were identified by the corresponding 6x6 and 3x3 mm optical coherence tomography angiography scans (middle and right panel).
