# Supplementary material for: The role of dual antiplatelets in geographic atrophy secondary to non-neovascular aged-related macular degeneration
Source: Front Ophthalmol (Lausanne). 2022 Sep 8;2:984903. doi: 10.3389/fopht.2022.984903 (PMC11182290; doi:10.3389/fopht.2022.984903)
Supplement: Supplementary file 2 [file DataSheet_2.pdf]

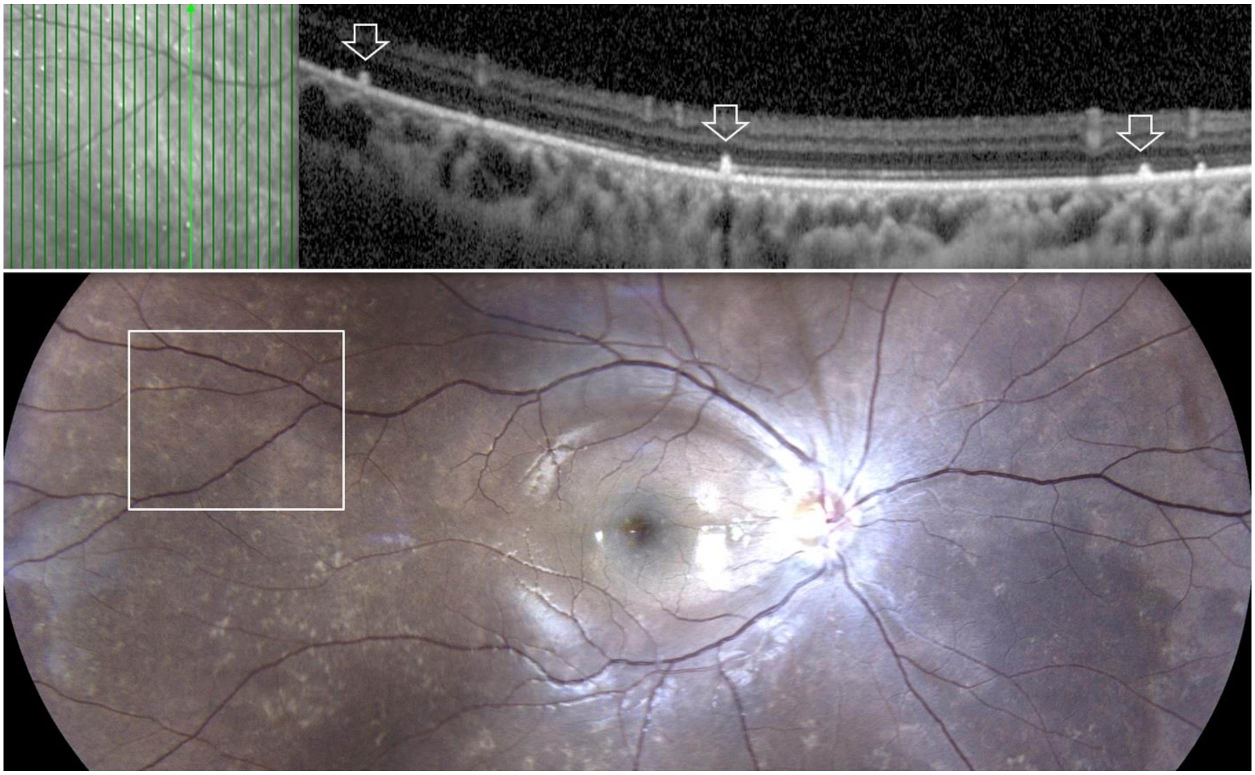

Supplementary Fig 2. Reticular pseudodrusen (RPD) developed after an episode of malignant hypertension. A generalized distribution of RPD confirmed by typical characteristics of subretinal drusenoid deposit (white arrows) on the OCT scans (white box) occurred in a patient with posterior reversible encephalopathy syndrome at one week after an episode of severe hypertensive retinopathy.
