# Supplementary material for: The role of dual antiplatelets in geographic atrophy secondary to non-neovascular aged-related macular degeneration
Source: Front Ophthalmol (Lausanne). 2022 Sep 8;2:984903. doi: 10.3389/fopht.2022.984903 (PMC11182290; doi:10.3389/fopht.2022.984903)
Supplement: Supplementary file 3 [file DataSheet_3.pdf]

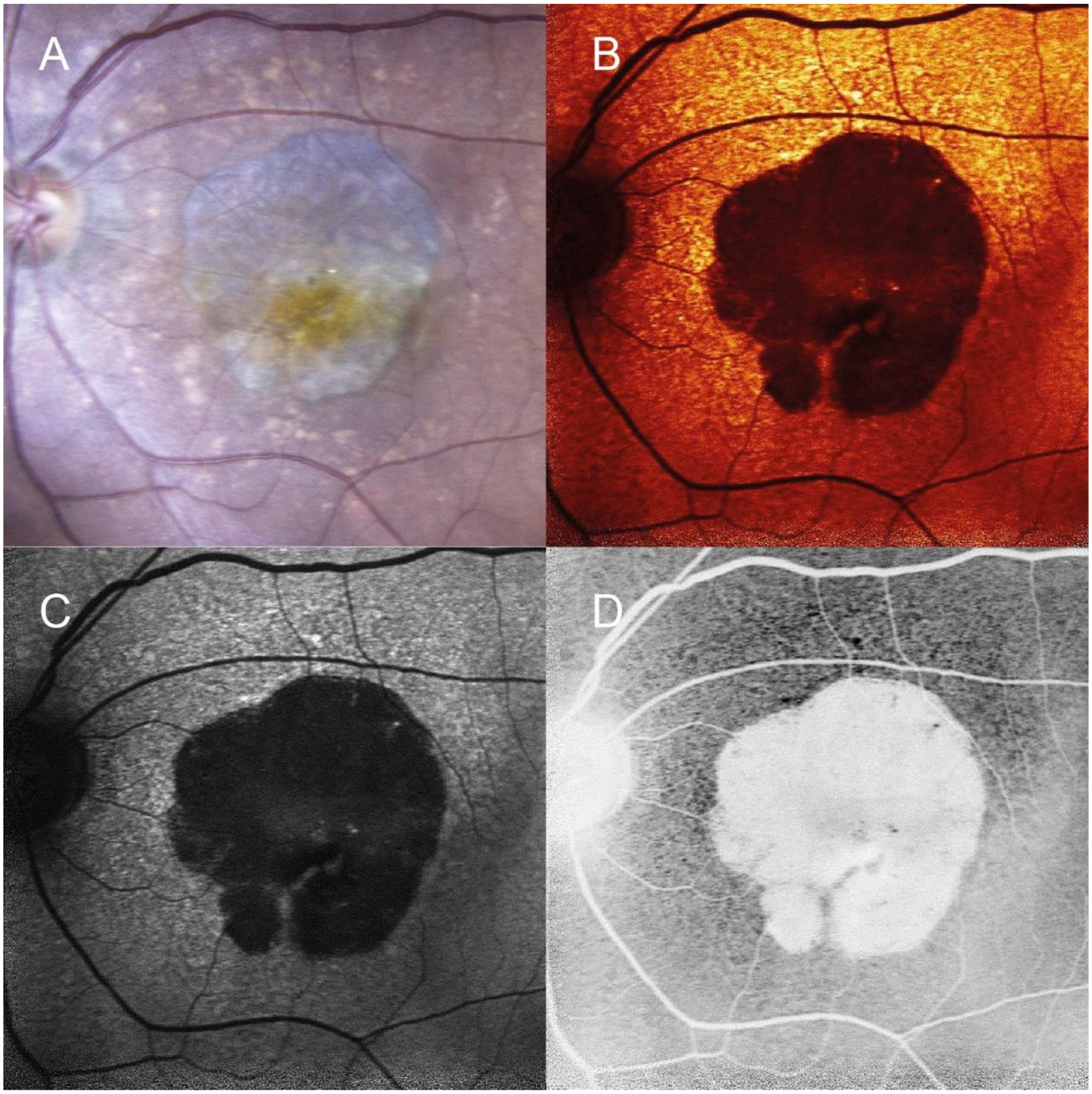

Supplementary Fig. 3. Color fundus photography **(A)** and fundus autofluorescence obtained from a patient with macular atrophy at the same visit. Color **(B)**, White-on-Black **(C)**, and Black-on-White FAF images are illustrated at the normal intensity setting. No additional image enhancement was performed.
