# Supplementary figures and images for: The role of dual antiplatelets in geographic atrophy secondary to non-neovascular aged-related macular degeneration
Source: Front Ophthalmol (Lausanne). 2022 Sep 8;2:984903. doi: 10.3389/fopht.2022.984903 (PMC11182290; doi:10.3389/fopht.2022.984903)

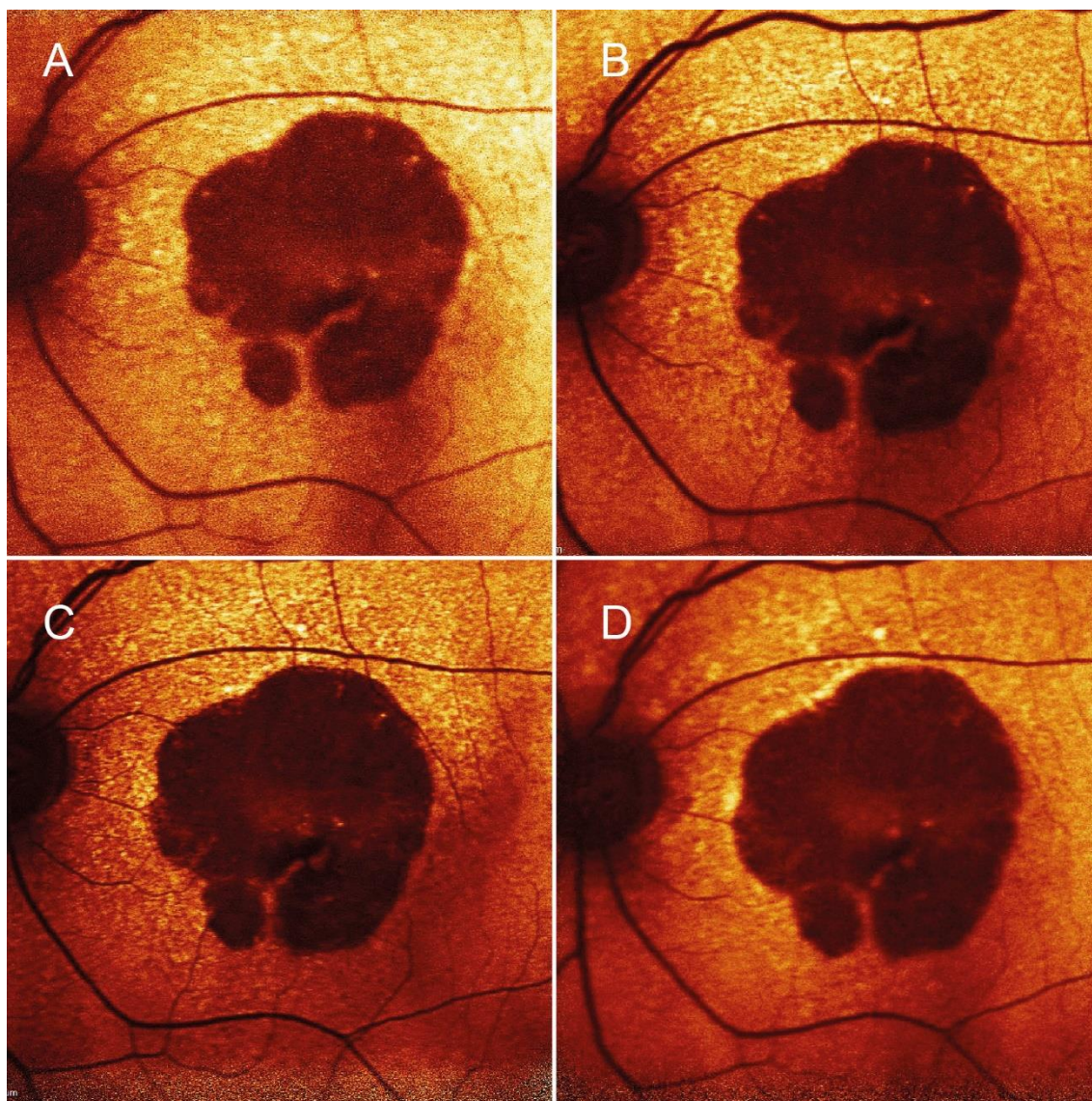

**Supplementary Fig. 4. Figure 1 without the measurement markers.**

Supplement: Supplementary file 4 [file DataSheet_4.pdf]
