# Supplementary material for: The role of dual antiplatelets in geographic atrophy secondary to non-neovascular aged-related macular degeneration
Source: Front Ophthalmol (Lausanne). 2022 Sep 8;2:984903. doi: 10.3389/fopht.2022.984903 (PMC11182290; doi:10.3389/fopht.2022.984903)
Supplement: Supplementary file 5 [file DataSheet_5.pdf]

Sample size calculation

We estimate mean square root of changes in macular atrophy of 0.28 mm/year based on a previous study.[1] Standard deviation of 0.13 is chosen based upon our preliminary data of the control group (mean, 0.19 mm/year), and we assume that the standard deviation will be the same in patients regularly taking dual antiplatelet therapy. Regarding most cut-off values reported from ongoing clinical trials involving GA treatment, we expect that dual antiplatelet therapy will decrease the progression rate by 20% over 12 months.[2] After completing calculations using the *sampsi* command in the STATA (as described below), we propose a sample size of 37 eyes per group to have 80% power to detect the proposed difference at the two-sided 0.05 level using a two-sample t-test.

power two means 0.28 0.22, sd*1(*0.13*)* sd*2(*0.13*)* alpha(*0.05)* power(*0.80)*

Estimated sample sizes for a two-sample means test

Satterthwaite's t test assuming unequal variances

Performing iteration

Ho: m2 = m1 versus Ha: m2 != m1

Study parameters: alpha = 0.0500; power = 0.8000; delta = -0.0600

m1 = 0.2800   m2 = 0.2200   sd1 = 0.090   sd2 = 0.0900

Estimated sample sizes:

N = 74, N per group = 37

REFERENCES

1. Chew EY, Clemons TE, Agron E, et al. Ten-year follow-up of age-related macular degeneration in the age-related eye disease study: AREDS report no. 36. JAMA Ophthalmol. 2014;132(3):272-7.
2. Halawa OA, Lin JB, Miller JW, Vavvas DG. A Review of Completed and Ongoing Complement Inhibitor Trials for Geographic Atrophy Secondary to Age-Related Macular Degeneration. J Clin Med. 2021;10(12).
